# *KaMRaT*: a C++ toolkit for *k*-mer count matrix dimension reduction

**DOI:** 10.1093/bioinformatics/btae090

**Published:** 2024-03-05

**Authors:** Haoliang Xue, Mélina Gallopin, Camille Marchet, Ha N Nguyen, Yunfeng Wang, Antoine Lainé, Chloé Bessiere, Daniel Gautheret

**Affiliations:** I2BC, Université Paris-Saclay, CNRS, CEA, 91190 Gif-sur-Yvette, France; I2BC, Université Paris-Saclay, CNRS, CEA, 91190 Gif-sur-Yvette, France; Univ. Lille, CNRS, Centrale Lille, UMR 9189 CRIStAL, F-59000 Lille, France; I2BC, Université Paris-Saclay, CNRS, CEA, 91190 Gif-sur-Yvette, France; I2BC, Université Paris-Saclay, CNRS, CEA, 91190 Gif-sur-Yvette, France; I2BC, Université Paris-Saclay, CNRS, CEA, 91190 Gif-sur-Yvette, France; IRMB, University of Montpellier, 34295 Montpellier, France; I2BC, Université Paris-Saclay, CNRS, CEA, 91190 Gif-sur-Yvette, France

## Abstract

**Motivation:**

*KaMRaT* is designed for processing large *k*-mer count tables derived from multi-sample, RNA-seq data. Its primary objective is to identify condition-specific or differentially expressed sequences, regardless of gene or transcript annotation.

**Results:**

*KaMRaT* is implemented in C++. Major functions include scoring *k*-mers based on count statistics, merging overlapping *k*-mers into contigs and selecting *k*-mers based on their occurrence across specific samples.

**Availability and implementation:**

Source code and documentation are available via https://github.com/Transipedia/KaMRaT.

## 1 Introduction

RNA-seq data analysis commonly involves comparison of sequence reads to a reference genome or transcriptome and quantification of annotated genes or transcripts (Van den Berge *et al.* 2019). While convenient, this approach ignores a wide range of variations present in the original sequence data. These variations may come from novel RNA isoforms, RNAs from repeats, intergenic regions and exogeneous species such as viruses, as well as from RNAs with small variations such as SNPs and indels. An emerging strategy to investigate all possible RNA variations at once is to use *k*-mers. First, a *k*-mer counter (e.g. Marçais and Kingsford 2011, Lemane *et al.* 2022b) extracts and counts all successive substrings of length *k* from the raw sequence reads. Various pipelines use *k*-mer count tables to select biologically relevant *k*-mers, possibly combining *k*-mers into longer contigs (Audoux *et al.* 2017, Rahman *et al.* 2018, Lorenzi *et al.* 2020, Lemane *et al.* 2022a). However, pipelines are cumbersome and slow to run. We felt that a standalone program performing common operations on *k*-mer count tables, such as *k*-mer selection and contig assembly would facilitate a greater diffusion of *k*-mer analysis among RNA-seq users. We thus developed *KaMRaT* (*k*-mer Matrix Reduction Toolkit), a lightweight and multi-functional toolkit implemented in C++ for *k*-mer matrix analysis, filtering and contig assembly.

## 2 Program description

**Figure 1.**
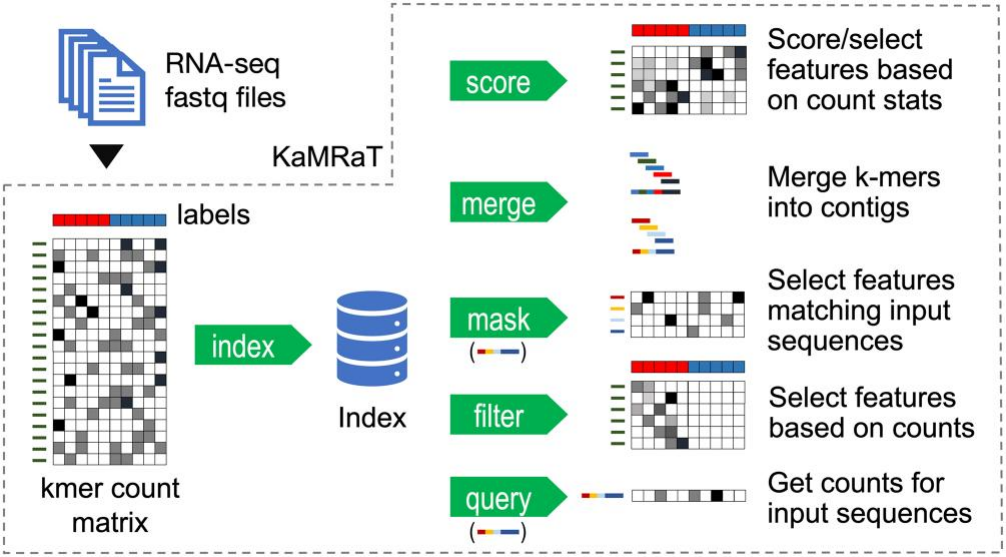
*KaMRaT* modules. Once an index is created, modules can be combined in different order, such as *score*+*merge*. Modules *mask* and *query* require a set of sequences as further input. Module *merge* can also output a new count table for the contigs.

*KaMRaT* takes as input a *k*-mer count table generated by any *k*-mer counter with *k*-mers as the first column. Other types of features are allowed (for instance gene IDs, sequence contigs) for certain operations, as long as they are also provided as the first column of the table. The program is composed of six main modules (Fig. 1 and Supplementary Methods).

- *index* creates a binary index of the features and counts on disk. This allows subsequent modules to randomly access count vectors without parsing the whole table.
- *score* scores and selects features based on univariate statistical tests, using categorical or numerical column labels provided as input. Available tests include T-test, signal-to-noise ratio (Golub *et al.* 1999), Detection of Imbalanced Differential Signal (DIDS) (de Ronde *et al.* 2013), logistic regression accuracy, Bayes classifier accuracy, Pearson and Spearman correlation. Label-free tests are also available, including absolute and relative standard deviation and information entropy.
- *merge* merges *k*-mers into contigs. By default, overlapping *k*-mers are iteratively merged until an ambiguity is met or no more *k*-mer satisfying a given minimum overlap length is available, similar to “unitigs” (Pevzner *et al.* 2004). An optional “intervention” mode uses correlation between count vectors to determine whether two *k*-mers or contigs with an acceptable overlap should be merged.
- *filter* deletes or selects features based on counts and recurrence thresholds.
- *mask* deletes or selects features matching given fasta sequences.
- *query* estimates count vectors of an input list of sequences based on their constitutive *k*-mers.

## 3 Performance overview

We assessed *KaMRaT* on simulated and real datasets of up to 150 RNA-seq samples. The index size is about 300 Gb for 150 samples (Supplementary Fig. S1A), i.e. equivalent to 1/3 of the original compressed fastq files. Once the index is created, *score* operations run in linear time and memory, allowing to process 420M *k*-mers or contigs in 85 min on a single processor with only 6 Gb RAM. The most ressource-intensive module, *merge* runs in log-linear time and can process 420M *k*-mers in about 3 h, using 80 Gb of RAM (Supplementary Fig. S1B). Note that large *k*-mer tables can be first processed with *score* so that only a subset of high scoring *k*-mers are fed to *merge*, thus considerably reducing the memory requirement.

A key contribution of *KaMRaT* is the intervention mode enabling a significant reduction of misassemblies when merging *k*-mers. Using *k*-mers from simulated reads or real RNA-seq reads from human tissues followed by T-test selection (Supplementary Material), the various intervention options changed 5%–25% of the output contigs compared to intervention-free *k*-mer extension (Supplementary Fig. S2A). Contigs produced using any of the intervention options were shorter but had higher rates of perfect alignment when aligned to reference transcripts (Supplementary Fig. S2B and C).

## 4 Applications

Typical *KaMRaT* applications are briefly presented below. Example workflows and a toy dataset are provided in supplementary material and the *KaMRaT* github repository https://github.com/Transipedia/KaMRaT/tree/master/toyroom. Although RNA-seq data are used in examples, the program can be applied to ChiP-seq or ribo-seq experiments as well.

### 4.1 Supervised feature selection

This is the first intended application of *KaMRaT*, typically achieved using an *index-score-merge* pipeline. *K*-mers of interest (for instance differentially expressed) are selected using any of the supervised test provided in the *score* module, and selected *k*-mers are merged into contigs.

### 4.2 Unsupervised feature selection

*KaMRaT score* supports unsupervised feature selection using standard deviation and information entropy. These methods can help reduce a *k*-mer table dimension independently of the variable to be predicted, thus avoiding information leakage in machine learning applications.

### 4.3 Finding features correlated to another feature

*KaMRaT score* enables retrieving features that correlate with a quantitative target vector, such as time, a measure of drug effect or the expression of a given gene.

### 4.4 Retrieving condition-specific *k*-mers or contigs

*KaMRaT filter* can be used to identify features expressed exclusively in samples from one condition. For instance, in a dataset with normal and tumor samples a *KaMRaT index-filter-merge* workflow can retrieve tumor-exclusive contigs.

## 5 Perspectives

*KaMRaT* offers a unique suite of tools for studying feature dimensionality and RNA variations. Three modules are pivotal: *score*, *merge*, and *filter*. *KaMRaT score* integrates currently 12 methods to reduce feature dimensionality in a supervised or unsupervised fashion, which should fit multiple research situations. *KaMRaT merge* builds on the concept of local *k*-mer extension (“unitigs”) to improve extension precision by leveraging count data. By our tests, intervention significantly improved extension correctness. However, these contigs do not compare to those produced by a full-length transcript assembler in that they are interrupted whenever ambiguities occur in the graph, for instance when encountering an SNP. *KaMRaT filter* allows retrieval of condition-specific features, which can be useful for collecting all RNA variations that are specific to a given sample set.

Although designed primarily for *k*-mer matrices, the *score* and *filter* modules apply to any generic count matrix such as gene-/transcript-expression matrices. This enables building classifiers from reference-free features (*k*-mers, contigs) and reference-based features (genes, transcripts) in a consistent and comparable way.

## Supplementary Material

btae090_Supplementary_Data
