# Supplementary material for: KaMRaT: a C++ toolkit for k-mer count matrix dimension reduction
Source: Bioinformatics. 2024 Mar 5;40(3):btae090. doi: 10.1093/bioinformatics/btae090 (PMC10942800; doi:10.1093/bioinformatics/btae090)
Supplement: btae090_Supplementary_Data [file btae090_supplementary_data.pdf]

Supplementary material for "*KaMRaT*: a C++ toolkit for k-mer count matrix dimension reduction", by Haoliang Xue et al.

Supplementary Methods

Workflow overview

KaMRaT's workflow can be described as follows: several samples (definition 1) are processed so that each feature (definition 4) is associated to a sample count vector (definition 5). The resulting feature count matrix (definition 6) is the input of the *KaMRaT* workflow. An index is then computed over the matrix, which can be processed through different operations. Thereby, the initial matrix is reduced into a sub-matrix where features are more relevant to a research goal, and - if features are k-mers (definition 2) - less interdependent. In a nutshell, given a dataset of which the count matrix has dimension $P \times N$, *KaMRaT* aims to reduce the matrix to dimension $p \times N$ where $p < P$.

Definitions

Definition 1: Sample and dataset. *A sample may correspond to one (in single-end sequencing) or two (in paired-end sequencing) FASTQ files. Multiple samples used in the same analysis compose a dataset. Let N denote the number of samples in the dataset for KaMRaT processing. Samples are numbered from 0 to $(N-1)$.*

Definition 2: k-mer and k-mer count. *k-mers are successive, overlapping substrings of a fixed length k extracted from sequencing reads. k-mer occurrences can be counted across samples, which are denoted by k-mer counts hereafter.*

Definition 3: k-mer merging/extension and k-mer contigs. *Merging and extension are two equivalent terms, describing the process of aggregating overlapping k-mers to produce a single, longer sequence, called a k-mer contig, or contig for short.*

Definition 4: Feature. *A feature is the abstraction of one property in a dataset. According to the context, a feature can denote a k-mer, a contig, a gene, or a transcript. Let P denote the number of features in the considered dataset.*

Definition 5: Sample count vector. *A sample count vector is a numeric vector of size N, which stores a vector of feature counts across samples $\{0, \dots, N-1\}$.*

Definition 6: Count matrix. *A count matrix results from stacking sample count vectors of multiple features, where the rows and columns represent features and samples, respectively.*

Indexation

To avoid repetitive parsing of the whole matrix by different modules, *KaMRaT* indexes the count matrix that is used for every further operation into binary index files, allowing random access to sample count vectors. Feature names/strings and count vectors are indexed into a single file, with the index positions being stored separately. Downstream modules then only rebuild in memory

the association between a feature and the indexed position to its sample count vector, avoiding repetitive processing of count vectors at each subsequent step. Optionally, users may normalize counts at this step based on total counts per sample, following equation (1):

$$X_{f,s}^{norm} \leftarrow \frac{X_{f,s}^{raw}}{\sum_{f_x \in \mathcal{F}} X_{f_x,s}^{raw}} \cdot C \tag{1}$$

where $X_{f,s}^{norm}$, $X_{f,s}^{raw}$: normalized and raw count of feature f and sample s; $\mathcal{F}$: universe of all features; C: constant scaling factor provided by user.

Indexation algorithm. *KaMRaT index* generates three files: (i) idx-meta.bin that stores meta-information, (ii) idx-mat.bin which encodes the count matrix, and (iii) idx-pos.bin comprising position for each row of the index matrix in idx-mat.bin, feature by feature. The meta-information includes: sample number, k-mer size, k-mer strandedness, header row of the input matrix and computed normalization factors. In the case of non-k-mer features, the k-mer size is set at 0 as a placeholder and strandedness information is ignored. The idx-mat.bin file encodes each feature with its count vector as a row. Each count vector is normalized (if required by user), converted to a character string by bit with *reinterpret_cast*, and written into the file followed by the corresponding feature's sequence/name. The idx-pos.bin file can be organized in two ways according to the value of k-mer size. If it equals to 0 (i.e., non-k-mer features), it contains a series of position value encoded into character by bit. Otherwise, if features are k-mers, it's composed by a series of pairs encoded from compressed k-mer integer code and the corresponding position. The compressed k-mer integer code is computed by mapping each nucleotide into two bits: $A = 00_2, C = 01_2, G = 10_2, T = 11_2$.

Note on k-mer size. The k-mer size is set by the k-mer counter and specified at the time of indexation. *KaMRaT* requires that k-mers be no longer than 32nt which is the maximum length that an int64 variable can store. There is no lower limit, however for specificity considerations, we recommand to use at least 15nt. Also short k-mers would inevitably lead to a higher rate of misassembly by *KaMRaT merge*. Finally, it is recommended to use an odd size number, to avoid potential confusions between a k-mer with its reverse-complement when k is even (e.g., AAATTT when $k = 6$).

Operations

Relying on the binary index of the matrix, *KaMRaT* can perform five operations.

- *score* scores features by their count vectors' dispersion, informativeness, or association to a given categorical/numerical vector;
- *merge* extends k-mers into longer sequences (contigs) based on sequence overlap;
- *filter* extracts/eliminates features according to their counts;
- *mask* retains/removes k-mers matching an input sequence list;
- *query* estimates count vectors of a given input list of sequences (k-mers or contigs) from the indexed count matrix.

KaMRaT score applies univariate feature scoring by different methods, with an option to select top figures for dimensionality reduction. It scores features either by estimating their count vector's association to a target vector of the same dimension, or by computing their count vector's dispersion or amount of information without involving any supplementary variable. The operation allows to select the top x or top $x\%$ features according to user's indication.

When a target vector is required for association estimation, it is provided through a list of $(sample, value)$ pairs in a tabular input file (design file), with the *value* being either categorical (such as samples' biological conditions) or numerical (such as samples' estimated immune cell abundance).

Table 1 summarizes available scoring methods with their acceptable target vector type. Detailed information on scoring methods are provided below in this document (*Details on scoring methods*).

Table 1: Scoring methods in *KaMRaT score*

scorer	target vector type	returned value
**ttest.padj**	categorical, 2 classes	t-test adjusted p-value with B-H procedure
**ttest.pi**	categorical, 2 classes	t-test π-value Xiao et al. (2014)
**snr**	categorical, 2 classes	signal-to-noise ratio Golub et al. (1999)
**dids**	categorical, ≥ 2 classes	DIDS de Ronde et al. (2013), adapted
**lr**	categorical, 2 classes	logit regression's accuracy
**bayes**	categorical, ≥ 2 classes	Bayes classifier's accuracy
**pearson**	numerical	Pearson correlation
**spearman**	numerical	Spearman correlation
**sd**	-	standard deviation
**rsd1**	-	relative standard deviation to mean count
**rsd2**	-	relative standard deviation to minimum count
**entropy**	-	information entropy

KaMRaT merge performs k-mer extension into *contigs* (see definition 3). k-mer contigs are obtained using a greedy extension procedure as in (Audoux et al., 2017), i.e., overlapping k-mers are iteratively merged until an ambiguity is met (multiple predecessors/successors) or no more k-mer satisfying a given minimum overlap length (by default $\lfloor k/2 \rfloor$nt) is available. This extension procedure is conservative : all bifurcations including those due to frequent mutations (such as SNV, indel, etc.) are considered as stop signs. This makes merging quite different from assembly methods such as *rnaSPAdes* (Bushmanova et al., 2019). In principle, this procedure is subject to extension errors, where k-mers from independent loci are merged due to their accidental overlap (which frequently occurs in repeats). This led us to propose a novel constraint for local extension: *disagreement of count vectors* (see definition 7).

Definition 7: Disagreement of count vectors. *The disagreement of two count vectors is a distance measure used to estimate how likely two count vectors are to be produced by different RNA transcripts.*

When using the *intervention* option of *KaMRaT-merge*, disagreement between count vectors is computed to determine whether two k-mers or contigs with an acceptable overlap should be merged.

Disagreement is estimated by either Pearson distance, Spearman distance or mean absolute contrast (MAC), defined by equation set (2) where all measures are scaled between 0 and 1. By default, extension is executed when $d_{\text{Pearson}} < 0.20$.

$$\begin{cases} d_x(\boldsymbol{c_1}, \boldsymbol{c_2}) = \frac{1}{2} \times (1 - \rho_x(\boldsymbol{c_1}, \boldsymbol{c_2})), & x = \text{Pearson}, \text{Spearman} \\ d_x(\mathbf{c_1}, \mathbf{c_2}) = \text{mean}_{s \in \mathcal{S}} \left(\left| \frac{c_{1,s} - c_{2,s}}{c_{1,s} + c_{2,s}} \right| \right), & x = \text{MAC} \end{cases} \tag{2}$$

where d_x: Pearson/Spearman/MAC distance; $\mathbf{c_1}$ and $\mathbf{c_2}$: sample count vectors of the two k-mers adjacent to the overlapping part, with $c_{1,s}$ and $c_{2,s}$ being the components of sample s; $\mathcal{S}$: universe of all samples; ρ_x: Pearson/Spearman correlation coefficient.

Definition 8: Stable merging. *A stable merging is achieved if the resulting contig is determined only by the information at either adjacent side of the overlapping part, independent from information on any other location.*

Stable merging guarantees obtaining the same results regardless of input order of k-mers. The original k-mer merging procedure, by only considering prefix-suffix overlap, achieves this by nature; however, with sample-count intervention, this does not hold true when count vectors of k-mers not located at the merging site are considered for disagreement evaluation. Therefore, to guarantee *stable merging*, disagreement is evaluated only between the two k-mers at the merging site.

After a merging operation, the count vector of the resulting contig can be computed for each sample as the mean or median counts of all constituent k-mers, according to user's preference.

KaMRaT filter filters features according to their abundance and recurrence in one or more samples. It can be used to select features expressed above a certain level in one or several samples. Users must provide a design file in which samples are labelled as either "UP" or "DOWN", and *KaMRaT filter* will select features such that :

$A(f, s) >= c_{up}$ in at least r_{up} "UP"-labelled samples
AND
$A(f, s) <= c_{down}$ in at least r_{down} "DOWN"-labelled samples

where $A(f, s)$ is the abundance of a feature f in sample s, and $c_{up}, c_{down}, r_{up}, r_{down}$ are provided by users.

A simple use case is the retrieval of features present exclusively in the case condition. Here, control samples are labelled as "DOWN", with $c_{down} = 0$ and $r_{down} = \#\{\text{control samples}\}$.

KaMRaT filter also supports the reverse operation, i.e., to remove the features satisfying given criteria rather than to select them.

KaMRaT mask retains or removes k-mers matching an input list of sequences. k-mers in the indexed matrix are retained or removed only if they have exact matches in the input sequence.

KaMRaT query estimates count vectors of an input list of sequences based on their constituent k-mer counts in the indexed matrix. The query operation has two modes : mean and median, which compute respectively the mean or median count vector of input sequences' constituent k-mers found in the index. If a sequence has no constituent k-mer found, it can be either omitted or returned with an all-zero vector, as per user's preference.

Workflow examples

KaMRaT's modular design allow users to quickly test and practice different workflows (Figure 1, main text). To produce the input k-mer count matrix from individual RNA-seq files, we provide a companion script using *Jellyfish* (Marçais and Kingsford, 2011) and *DE-kupl joinCounts* (Audoux et al., 2017) (`https://github.com/Transipedia/KaMRaT/tree/master/related-tools`).

The *KaMRaT* repository includes different workflow examples and a toy dataset (`https://github.com/Transipedia/KaMRaT/tree/master/toyroom/`) composed of 89,768 k-mers $\times$ 20 samples from 10 lung tumor and 10 normal lung samples from a public dataset (Seo et al., 2012) (kmer-counts.subset4toy.tsv.gz), and a (sample, condition) file (sample-condition.toy.tsv).

Typical use cases with these data include:

```
# Always Index as first step
kamrat index -intab kmer-counts.subset4toy.tsv.gz -outdir kamrat.idx \
             -klen 31 -unstrand -nfbase 1000000000

# Case 1: merge k-mers into contigs, then select top 5% contigs
#         by t-test p-values between conditions.
kamrat merge -idxdir kamrat.idx -outpath merged-kmers.bin
kamrat score -idxdir kamrat.idx -scoreby ttest.padj -design sample-condition.toy.tsv \
             -seltop 0.05 -with merged-kmers.bin -outpath top-ctg-counts.tsv -withcounts

# Case 2: score k-mers by Spearman correlation to a value vector,
#         then merge the top 5000 k-mers into contigs.
kamrat score -idxdir kamrat.idx -scoreby spearman -design sample-values.toy.tsv \
             -seltop 5000 -outpath top-kmers.bin
kamrat merge -idxdir kamrat.idx -overlap 30-15 -with top-kmers.bin:min \
             -outpath top-ctg-counts.tsv -withcounts mean

# Case 3: fetch k-mers with >= 3 counts in >= 5 "up" samples
#         and zero counts in "down" samples, as labelled in an external list,
#         then merge them into contigs.
kamrat filter -idxdir kamrat.idx -design sample-up.toy.tsv \
              -upmin 3:5 -downmax 0:10 -outpath top-kmers.bin
kamrat merge -idxdir kamrat.idx -overlap 30-15 -with top-kmers.bin:min \
             -outpath top-ctg-counts.tsv -withcounts mean

# Case 4: fetch k-mers absent in a reference (novel k-mers)
          then merge them into contigs.
kamrat mask -idxdir kamrat.idx -fasta gencode.v34.fa -outpath masked-kmers.bin
kamrat merge -idxdir kamrat.idx -overlap 30-15 -with masked-kmers.bin:min \
             -outpath novel-ctg-counts.tsv -withcounts mean

# Case 5: obtain counts for sequences in set, computed as median
#         of component k-mer counts.
kamrat query -idxdir kamrat.idx -fasta sequences.fa -toquery median \
```

```
            -outpath query-ctg-counts.tsv
```

As an example, the first case reduces the table from 89,768 k-mer features to 4,792 contig features, among which 68 have a t-test adjusted p-value < 0.05. Using the -seltop option provided by *KaMRaT score*, users can keep for example top 5% contigs (i.e., 240) regardless of their p-value for further investigation.

Dependencies

KaMRaT is implemented in C++ with dependencies on *MLpack* (Curtin et al., 2018), *Armadillo* (Sanderson and Curtin, 2016) and *Boost Iostreams* (Schäling, 2011) libraries. Source code is available on a GitHub repository `https://github.com/Transipedia/KaMRaT`.

Benchmarking

Hardware. Benchmarks were performed on a PBS cluster using a single node CPU (Intel Xeon E5-2640v4 @ 2.40GHz, 750Gb memory) with network-mounted hard disk drive (I/O times clocked at 258MB/s).

D1 - synthetic error-free RNA-seq reads. This dataset contains 20 samples, synthesized based on Gencode transcripts longer than 5500 nt. We applied *Mutation-Simulator* (Kühl et al., 2021) on this reference to create 10 individual transcriptomes with mutations and applied the *polyester* R package (Frazee et al., 2015), requesting error-free sequence reads with a sequencing depth of 0.2 to emulate discontinuous coverage.

D2 - real RNA-seq dataset. This dataset is composed of 154 lung adenocarcinoma RNA-seq samples (77 tumors and 77 adjacent normal tissues) from SRA accession ERP001058 (Seo et al., 2012), processed by *Cutadapt* (Martin, 2011) for adapter and low-quality bases trimming.

k-mer count matrices. The k-mer count matrices for datasets **D1** (149M k-mers) and **D2** (484M k-mers) were produced using our script `https://github.com/Transipedia/KaMRaT/tree/master/related-tools/prepare_kmer_table`. For dataset **D2**, subsets corresponding of 5 to 100 samples were produced by selecting 5 to 100 columns in the larger matrix and deleting k-mers with zero sum counts.

Benchmark of *KaMRaT* for *index*, *merge* and *score*

Runtime, memory and disk usage were first evaluated on k-mer matrices from real dataset **D2** with 5 to 154 samples (Figure S1A). However in this test, both the number of k-mers and the number of counts for each k-mer increases with the number of samples. To observe the effect of the number of k-mers or features alone, we took the **D2** matrix for 50 samples and downsampled it keeping 3.5M to 420M k-mers. This allowed us to observe CPU time and RAM usage as a function of the number of k-mers or features, at a constant number of samples (Figure S1B).

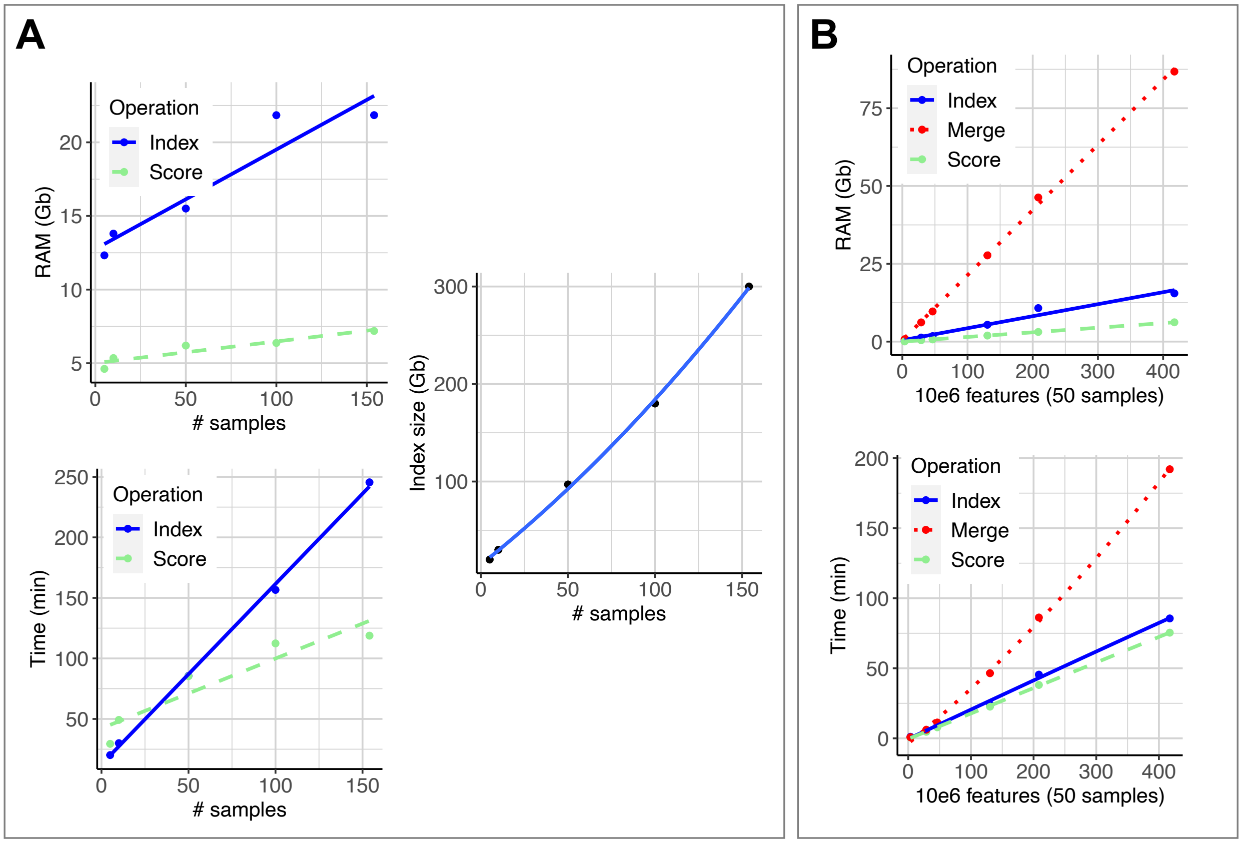

Figure S1: Memory, disk and CPU usage of the main *KaMRaT* modules. A: CPU time, memory and disk usage for *index* and *score* as a function of the number of samples in the **D2** real RNA-seq dataset. B: CPU time and memory usage for *index*, *score* and *merge* as a function of the number of k-mers (features) in the **D2** real RNA-seq dataset.

Evaluation of *merge* intervention modes

To assess the correctness of *KaMRaT merge* contigs, we used (i) the synthetic dataset **D1** where all reads were produced from a known set of transcripts and (ii) the real dataset **D2** restricted to 50 samples and further processed by *KaMRaT score* (T-test, "tumor" *vs.* "normal") keeping the top 1 million k-mers scored by T-test P-value. This last test emulated a typical situation where contigs are produced from a condition-specific list of k-mers.

KaMRaT index-merge was run with different intervention options (none and each intervention method: Mac:0.2, Pearson:0.2, Spearman:0.2). We then compared contigs produced by the different intervention options using Venn diagrams (Figure S2A. Next, we evaluated the correctness of contigs that were specific to each intervention method, by aligning contigs using *BLASTn* (Camacho et al., 2009) to either the transcript set used for simulating reads (simulated dataset **D1**) or to Gencode V34 transcripts (real dataset **D2**). Using *BLASTn* results, we measured the **perfect match ratio** as the percentage of contigs that were completely and exactly aligned - from first to last nucleotide, without any gap or mismatch - to a transcript in the reference (Figure S2B). Note that the lower

perfect match ratio in the real dataset is expected as many contigs correspond to transcripts that are not in Gencode or contain polymorphisms or sequencing errors. Finally, Figure S2C shows the average contig lengths for each category of option-specific contigs.

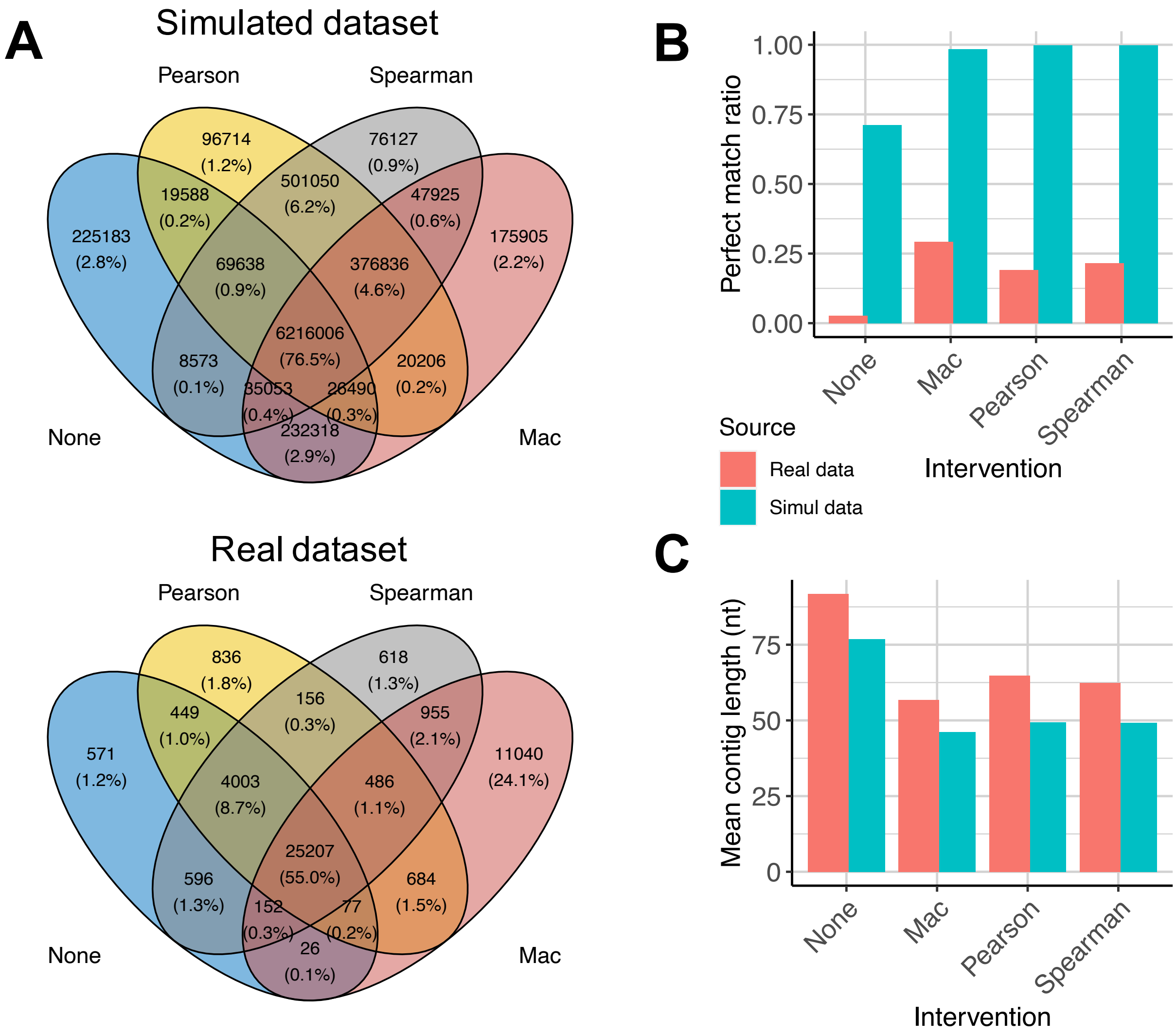

Figure S2: Effect of *merge* intervention modes. A: Venn diagram of contigs produced by the different intervention options, applied to the simulated (**D1**) and real (**D2**, subsampled) datasets. B: Perfect match ratio (contig perfectly aligned to reference over the contig length) of contigs identified specifically by each intervention option. C: Average length of contigs identified specifically by each intervention option.

Details on scoring methods

The features are scored by different methods in *KaMRaT score* and selected as described below:

ttest.padj scores features according to a binary label. Firstly a $\log_2(x+1)$ transformation is applied to sample counts. Then each feature association between sample counts and labels is evaluated by p-value t-test, adjusted by Benjamini-Hochberg procedure for controlling false discovery rate. The top features are selected from the lowest value to the highest.

ttest.pi scores features according to a binary label. It is calculated with the formula given in (Xiao et al., 2014) as shown in equation 3. The top features are selected from the highest value to the lowest.

$$\pi = -\log_{10}(p) \times |mean_{i\in G_1}[\log_2(S_i+1)] - mean_{j\in G_2}[\log_2(S_j+1)]| \tag{3}$$

where p is the non-adjusted p-value; G_1 and G_2 are two sample groups; S_i and S_j are counts of two samples from G_1 and G_2, respectively.

snr scores features according to a binary label. It is calculated by dividing the difference between group means by the sum of group standard deviations, followed by what proposed in (Golub et al., 1999), as shown in equation 4. The top features are selected with absolute values from highest to lowest.

$$snr = \frac{|\text{mean}_{i\in G_1}(S_i) - \text{mean}_{j\in G_2}(S_j)|}{\text{SD}_{i\in G_1}(S_i) + \text{SD}_{j\in G_2}(S_j)} \tag{4}$$

where G_1 and G_2 are two sample groups; S_i and S_j are counts of two samples from G_1 and G_2, respectively.

dids scores features according to a multiple-condition label. We generalized the initial case-vs-control version proposed in (de Ronde et al., 2013) to multiple conditions, by iteratively estimating the DIDS score under "one-group-vs-others" fashion and returning the maximum value as the final score, as shown below. The top features are selected from the highest to the lowest value.

$$\begin{aligned} \hat{S}_g &= max_{i\in g}(S_i) \\ dids &= max_g\left(\sum_{j\notin g}\sqrt{|S_j - \hat{S}_g|^+}\right) \end{aligned} \tag{5}$$

where g is a group label; S_i is the count of sample i; $|x|^+$ is x if $x > 0$ or 0 otherwise.

lr scores features according to a binary label. It estimates classification accuracy by logistic regression, calculated by *MLPack* (Curtin et al., 2018) library. The "lr" scoring method applies a standardization preprocessing of feature counts, i.e., substract all components of the sample count vector with their mean value, and divide them by the standard deviation. The top features are selected from highest to lowest value.

bayes scores features according to a multiple-condition label. It estimates classification accuracy using a Bayes classifier from the *MLPack* (Curtin et al., 2018) library. The "bayes" scoring method applies also a standardization preprocessing to feature counts. The top features are selected from highest to lowest score.

pearson and **spearman** score features using a vector of continuous values across samples (target numerical vector). These estimate Pearson or Spearman correlation between each feature's sample count vector and the given target numerical vector, followed by calculation of the absolute value. This option finally selects the top features ranked from highest to lowest score.

sd scores features without considering labels (i.e., non-supervised feature selection). It estimates each features' standard deviation across samples, and selects them from highest to lowest score.

rsd1 and **rsd2** score features without considering labels (i.e., non-supervised feature selection). They estimate each features' relative standard deviation across samples to the mean sample count (i.e., SD divided by mean value) or to the minimum sample count (i.e., SD divided by minimum value), respectively. Please note that when the mean or minimum count is less than 1, the original SD is returned without scaling. The methods select top features ranked from highest to lowest score.

entropy scores features without considering labels (i.e., non-supervised feature selection). It estimates each features' Shannon entropy across samples. In order to eliminate the interference of the 0 component in the vector, we added an offset 1 to each component of the sample count vector before calculating the entropy score. This method is used in the *iMOKA reduce* step for informative feature filtering, though *iMOKA* did not add the offset to the count vector, but skipped the 0 components. (Lorenzi et al., 2020). The top features are selected from smallest to largest score.

$$
\begin{aligned}
T &= \sum_{s \in \mathcal{S}} (c_s + 1) \\
\text{entropy} &= -\sum_{s \in \mathcal{S}} \left[\frac{c_s + 1}{T} \cdot \log_2 \left(\frac{c_s + 1}{T} \right) \right]
\end{aligned} \tag{6}
$$

where $\mathcal{S}$ is the sample set, c_s is the count of sample s.

Comparison of scoring methods. To compare *score* methods, we generated four feature count matrices for 300 samples and 20,000 to 200,000 features, using the *compcodeR* R package (Soneson, 2014). The four matrices differ from each other by the level of differential expression (DE) signal intensities and noises, as indicated in Figure S3A. Using the simulated DE status of features as ground truth, we evaluated the DE detection capability of each scoring method applicable to categorical variables, i.e., t-test adjusted p-value and π-value, SNR, DIDS, logistic regression and Bayes classifier, using precision-recall curves (Figure S3B). DIDS predictions were most distant from other scoring methods (Figure S3C). T-test adjusted p-values and π-values achieved the best performance, with respective PR AUCs of 0.794 and 0.785 in the most complicated case. SNR also showed favorable performance with a worst-case PR AUC of 0.637. These three methods estimate the difference between groups based on means and standard deviations, which coincides with how *compcodeR* simulates DE features. While other methods (Bayes, DIDS and LR) did not perform as well, it should be noted that Bayes and LR are not intended to detect DE features. In principle DIDS detects DE features by considering outlier observations, however the way outliers are generated in *compcodeR* is independent from differential feature labeling, thus it is of no use for DIDS, which probably explains its behavior.

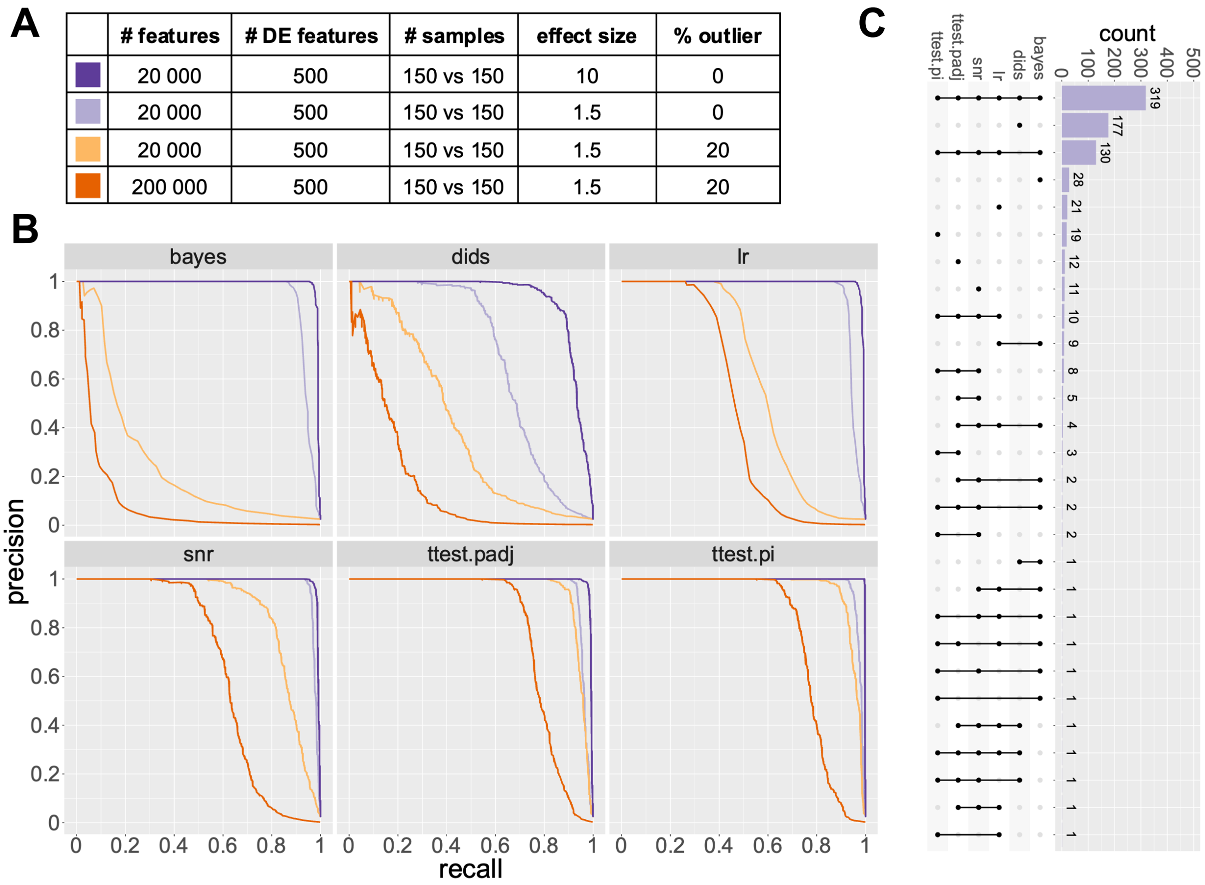

Figure S3: Comparison of *score* methods (color codes are defined in panel A). A: Characteristics of simulated count matrices. B. Precision-recall curves for classification of DE features by each scoring method. C. UpSetR plot comparing predicted DE features from each method, for the second simulated dataset (20,000 features, effect size 1.5, no outlier).

References

Audoux, J., Philippe, N., Chikhi, R., Salson, M., Gallopin, M., Gabriel, M., Le Coz, J., Drouineau, E., Commes, T., and Gautheret, D. (2017). De-kupl: exhaustive capture of biological variation in rna-seq data through k-mer decomposition. Genome biology, 18(1):243.

Bushmanova, E., Antipov, D., Lapidus, A., and Prjibelski, A. D. (2019). rnaspades: a de novo transcriptome assembler and its application to rna-seq data. GigaScience, 8(9):giz100.

Camacho, C., Coulouris, G., Avagyan, V., Ma, N., Papadopoulos, J., Bealer, K., and Madden, T. L. (2009). Blast+: architecture and applications. BMC bioinformatics, 10(1):421.

Curtin, R. R., Edel, M., Lozhnikov, M., Mentekidis, Y., Ghaisas, S., and Zhang, S. (2018). mlpack 3: a fast, flexible machine learning library. Journal of Open Source Software, 3:726.

de Ronde, J. J., Rigaill, G., Rottenberg, S., Rodenhuis, S., and Wessels, L. F. (2013). Identifying subgroup markers in heterogeneous populations. Nucleic acids research, 41(21):e200–e200.

Frazee, A. C., Jaffe, A. E., Langmead, B., and Leek, J. T. (2015). Polyester: simulating rna-seq datasets with differential transcript expression. Bioinformatics, 31(17):2778–2784.

Golub, T. R., Slonim, D. K., Tamayo, P., Huard, C., Gaasenbeek, M., Mesirov, J. P., Coller, H., Loh, M. L., Downing, J. R., Caligiuri, M. A., et al. (1999). Molecular classification of cancer: class discovery and class prediction by gene expression monitoring. science, 286(5439):531–537.

Kühl, M., Stich, B., and Ries, D. (2021). Mutation-simulator: fine-grained simulation of random mutations in any genome. Bioinformatics, 37(4):568–569.

Lorenzi, C., Barriere, S., Villemin, J.-P., Bretones, L. D., Mancheron, A., and Ritchie, W. (2020). imoka: k-mer based software to analyze large collections of sequencing data. Genome Biology, 21(1):1–19.

Martin, M. (2011). Cutadapt removes adapter sequences from high-throughput sequencing reads. EMBnet. journal, 17(1):10–12.

Marçais, G. and Kingsford, C. (2011). A fast, lock-free approach for efficient parallel counting of occurrences of k-mers. Bioinformatics, 27(6):764–770.

Sanderson, C. and Curtin, R. (2016). Armadillo: a template-based c++ library for linear algebra. Journal of Open Source Software, 1(2):26.

Schäling, B. (2011). The boost C++ libraries. Boris Schäling.

Seo, J.-S., Ju, Y. S., Lee, W.-C., Shin, J.-Y., Lee, J. K., Bleazard, T., Lee, J., Jung, Y. J., Kim, J.-O., Shin, J.-Y., et al. (2012). The transcriptional landscape and mutational profile of lung adenocarcinoma. Genome research, 22(11):2109–2119.

Soneson, C. (2014). compcoder—an r package for benchmarking differential expression methods for rna-seq data. Bioinformatics, 30(17):2517–2518.

Xiao, Y., Hsiao, T.-H., Suresh, U., Chen, H.-I. H., Wu, X., Wolf, S. E., and Chen, Y. (2014). A novel significance score for gene selection and ranking. Bioinformatics, 30(6):801–807.
